# Mediating effects of healthy lifestyle factors on associations between mental health and functional outcomes in early adolescence

**DOI:** 10.1017/S0033291726105121

**Published:** 2026-07-07

**Authors:** Jason Smucny, Tyler A. Lesh, Tara A. Niendam, Nicole R. Karcher

**Affiliations:** 1 Department of Psychiatry and Behavioral Sciences, University of California, Davis, Sacramento, USA; 2 Department of Psychiatry, Washington University in St Louis, USA

**Keywords:** adolescence, anxiety, depression, mediation, psychosis, screen time, sleep

## Abstract

**Background:**

Although mental health and healthy lifestyle interventions are associated with functional outcomes in adolescence, the extent to which particular lifestyle factors explain relationships between mental health and outcome are unclear. Here we examined mediating effects of lifestyle factors on relationships between mental health and two functional outcomes measured 2–3 years later, as well as moderating effects of environmental risk factors on mediation strength in early adolescence.

**Method:**

We analyzed data from three waves of the Adolescent Brain Cognitive Development Study (ages 10–11, 11–12, and 12–13 years). Mediating effects of sleep quality, screen time, physical activity, and Mediterranean diet on the relationships between depression, anxiety, psychotic-like experience (PLE) distress, and total problems with two subsequent functional outcomes (academic functioning and social problems) were examined. Secondary analyses included environmental factors as moderators.

**Results:**

Sleep quality mediated 18.5%, 36.3%, and 8.3% of the relationships between depression, anxiety, and PLE distress with academic functioning, respectively (total problems mediation was nonsignificant). Screen time was the second strongest mediator. For social problems, only sleep quality showed >3% mediation (19.6–23.3%). Mediating effects of sleep and screen time on academic functioning decreased as financial adversity increased. Conversely, mediating effects of sleep quality on social problems increased with worsening family conflict, financial adversity, and school environment.

**Conclusions:**

These results suggest that healthy lifestyle factors (particularly sleep quality) may partially explain associations between mental health and functioning in adolescents and suggest that these effects are modulated by environmental factors. These results may have implications for future intervention studies.

## Introduction

Adolescence represents a critical developmental window during which mental health concerns, including depression, anxiety, and psychotic-like experiences (PLEs), often emerge (Barch et al., 2018; Galvan, 2017; Olfson, Druss, & Marcus, 2015). Globally, approximately one in seven 10- to 19-year-olds experiences a mental disorder, and depression and anxiety are the leading causes of illness and disability among young people (Carvajal, Requejo, & Irwin, 2021; Remschmidt & Belfer, 2005). Understanding both the functional correlates of early mental health concerns and the targetable associative factors underlying their development is of great importance for both public health and developmental science. The current article directly addresses this need by examining potentially targetable mediating factors linked to the emergence of early mental health concerns.

Mental health difficulties experienced in adolescence often have a number of downstream consequences, including impact on everyday functioning such as academic achievement and peer/social functioning (Achenbach & Rescorla, 2001; Karcher et al., 2018). Mood and anxiety disorders have been associated with school absenteeism, lower grades, and withdrawal from peer relationships (Achterbergh et al., 2020; Dalforno, Wengert, Kim, & Jacobsen, 2022; Finning et al., 2019a, 2019b; Kearney, Dupont, Fensken, & Gonzálvez, 2023; Kupferberg & Hasler, 2023). Likewise, psychotic-like experiences (PLEs) may disrupt social engagement, peer support, and academic performance (El Bouhaddani et al., 2018; Huckle, Lemmel, & Johnson, 2021; Steenkamp et al., 2021). Failure to examine functioning alongside symptomatology risks overlooking the real-world consequences of adolescent mental health concerns and misses opportunities to intervene early and prevent these potential downstream impacts.

Given the relationship between early mental health concerns and everyday functioning, elucidating the pathways underlying these associations becomes imperative. Among readily modifiable associative factors, lifestyle aspects, including healthy sleep, physical activity, diet quality, and screen time, hold promise as leverageable targets in prevention and intervention. Research in ‘lifestyle psychiatry’ has underscored that physical activity, sleep, and diet contribute meaningfully to the onset, maintenance, and alleviation of mental disorders (Firth et al., 2020). For example, higher levels of exercise have been linked to reduced depressive and anxiety symptoms in youth, and emerging evidence also points to benefits for social and academic functioning (Ruiz-Ranz & Asin-Izquierdo, 2025). Sleep disruptions, including but not limited to insufficient duration, poor quality, or irregular timing, have been associated with increased internalizing symptoms and poorer school and interpersonal functioning in adolescents (Bruni et al., 1996; Nagata, Bashir, et al., 2024). Diet quality, especially adherence to a Mediterranean diet, has been connected to lower levels of depressive and anxiety symptoms in children and adolescents (Camprodon-Boadas et al., 2025; O’Neil et al., 2014). Screen time presents another critical lifestyle parameter: extensive screen exposure, especially via social media and passive screen use, has been consistently tied to worse mental health outcomes in adolescents, and may further impair social and academic engagement through sleep displacement, reduced activity, and diminished in-person peer interaction (Santos et al., 2023; Zablotsky et al., 2025). Consistent with this, research using the Adolescent Brain Cognitive Development (ABCD) study has found evidence that increased screen time (Nagata, Al-Shoaibi, et al., 2024; Zink et al., 2024), greater sleep disturbances (Goldstone et al., 2020; Reeve & Bell, 2023), and lower physical activity (Damme et al., 2024) are associated with greater endorsement of mental health concerns. The current study aims to examine the untested model that these lifestyle behaviors act as mediators that link adolescent mental health (e.g. depression, anxiety, and PLEs) to everyday academic and social functioning.

It is also likely that these lifestyle factors (i.e. sleep, physical activity, diet, and screen time) do not operate uniformly across all youth but rather are shaped by environmental context. For instance, environmental risk factors including financial adversity (e.g. socioeconomic strain), neighborhood and school conditions, and/or familial conflict may moderate the strength of the pathways linking lifestyle behaviors to mental health and functioning. Adolescents growing up in economically disadvantaged conditions (Aliyas, Mahmoudian, & Cloutier, 2025; Gautam, Dessie, Rahman, & Khanam, 2023; Gautam, Rahman, & Khanam, 2025; Steare et al., 2024) or high-conflict homes (Al-Shoaibi et al., 2024; Cummings, Koss, & Davies, 2015) may face compounding risk, such that the same amount of sleep disruption or elevated screen time may confer greater detriment in the presence of environmental risk factors, or lifestyle buffers such as exercise may be undermined by environmental risk factors. The possibility of moderated mediation, whereby the environment (e.g. family conflict and disadvantage) influences the degree to which lifestyle factors mediate associations between symptoms with functioning, is an unexplored yet potentially compelling framework for understanding heterogeneity in adolescent risk for functioning decline.

In the present study, we leveraged data from the longitudinal, multisite ABCD Study to test a conceptual model whereby adolescent depression, anxiety, and PLEs predict academic and social functioning, with lifestyle behaviors (screen time, sleep quality, physical activity, and diet) acting as mediators partially linking these associations. Although the mediating effects of many interventions could be examined, we focused on these four lifestyle factors not only because of their established links to functioning but also because they can be addressed in outpatient therapeutic settings. Follow-up analyses explored the possibility of moderated mediation, exploring whether links between lifestyle factors and functioning were moderated by environmental risk factors (i.e. financial adversity, neighborhood safety, school quality, and familial conflict). This work aims to provide novel evidence identifying actionable targets for early intervention and to delineate under which conditions healthy lifestyle factors are most impactful.

## Methods

### Participants

ABCD data used in this report came from release 5.1 (DOI: 10.15154/z563-zd24). DOIs can be found at https://nda.nih.gov/abcd/abcd-annual-releases.html. The present study primarily used 1-year follow-up data (participant ages 10–11 years) as clinical predictors, 2-year follow-up data (ages 11–12 years) as mediators (except for Mediterranean diet, which was only available at 1-year follow-up), and 3-year follow-up data (ages 12–13 years) as the outcome measure in mediation models. Potential participants were excluded from ABCD study participation for the following reasons: child not fluent in English, major neurological disorder, gestational age <28 weeks or birthweight <1,200 grams, history of traumatic brain injury, or has a current diagnosis of schizophrenia, autism spectrum disorder (moderate and severe), mental retardation/intellectual disability, or alcohol/substance use disorder. For families with siblings, only one sibling from the family was included (based on alphabetical order from the Global Unique Identifier).

### Mental health predictors

The following mental health measures were included as predictors in mediation models: Child Behavior Checklist (CBCL) (T.M. Achenbach & Rescorla, 2001) Depression T-score, CBCL Anxiety T-score, CBCL Total Problems T-score (a composite of all CBCL ratings), and Prodromal Questionnaire-Brief Child Version (PQ-BC) PLEs distress score sum (Karcher et al., 2018). Depression and anxiety scores were taken from the DSM-5-oriented affective problems and anxiety problems CBCL scales. All predictive clinical measures were taken from 1-year follow-up data.

### Healthy lifestyle mediators

The following ‘healthy lifestyle’ measures were included as potential mediators in analyses: diet, physical activity, screen time, and sleep quality. ‘Diet’ was captured by the parent-reported sum of the Mediterranean-DASH Intervention for Neurodegenerative Delay (MIND) diet questionnaire (see Supplementary Material) (Nagata, Bashir, et al., 2024). ‘Physical activity’ was captured by the child’s answer to the question: ‘During the past 7 days, on how many days were you physically active for a total of at least 60 minutes per day?’ (from 0 to 7). ‘Screen time’ was captured by parent-reported screen time on weekends or weekdays. Weekend and weekday screen time were examined separately because they may reflect different aspects of time allocation (i.e. recreational time allocation vs. educational [‘occupational’] time allocation). ‘Sleep quality’ was captured by taking the caregiver-reported sum of the Sleep Disturbance Scale for Children (SDSC) (Bruni et al., 1996) (see Supplementary Material). Screen time and sleep quality measures were reverse-coded such that greater values on all scales corresponded to greater adherence to following healthy lifestyle habits. Physical activity, screen time, and sleep quality were taken from 2-year follow-up data. Diet information was taken from 1-year follow-up data (as it was not included as part of 2-year follow-up data; notably, year-to-year diets are unlikely to substantially change between ages 10 and 12 (da Costa, Severo, Araujo, & Vilela, 2024).

### Functional outcomes

Two functional outcomes were examined: academic functioning during follow-up years 2–3, and social problems at 3-year follow-up (i.e. between ages 12 and 13). Parents provided academic functioning information at 3-year follow-up by reporting their child’s average grades during the preceding year converted to a 1–12 scale, with 1 corresponding to ‘A+’ or 97–100%, 2 with ‘A’ or 93–97%, 3 with ‘A− or 90–92%, and so forth, down to 12 with ‘F’ or < 65%. These scores were reverse-coded such that higher scores were associated with higher grades. Children whose parents refused to report grades were excluded from any analyses with grades. Social problems were quantified by CBCL Social Problems T-score, which includes questions regarding getting along with other children, getting teased, feeling lonely, and acting immaturely (Achenbach & Ruffle, 2000). Parents reported the grades students received over the course of 1 year, between the beginning of the ABCD study 2-year follow-up time point up to the beginning of the 3-year follow-up time point; thus, there was no temporal overlap with the lifestyle mediators collected at 2-year follow-up.

### Environmental adversity moderators

The following environmental adversity moderators were examined: neighborhood safety, family conflict, school environment, and family financial adversity. Neighborhood safety was captured by the parental report ABCD Neighborhood Crime and Safety Survey (Echeverria, Diez-Roux, & Link, 2004) (lower scores = safer neighborhood), family conflict by the ABCD Parent Family Environment Scale-Family Conflict Subscale (modified from PhenX; Moos, 1994) (higher scores = more conflict), school environment by the youth report School Risk and Protective Factors School Environment subscale (modified from PhenX; Zucker et al., 2018) (higher scores = better environment), and family financial adversity by the summed endorsement of seven parent-reported questions of financial difficulties experienced during the past 12 months from a demographic questionnaire (Karcher et al., 2020) (higher scores = more adversity). More detailed information on these instruments is provided in the Supplementary Material.

### Mediation analyses

Mediation analyses were performed using the PROCESS v.5.0 toolbox (processmacro.org) in SPSS v.30 (IBM). The primary analyses included eight mediation models; each model included one of the mental health measures (e.g. depression) as the independent variable, age/sex/site as covariates, the four healthy lifestyle factors (e.g. sleep quality) as simultaneous (parallel) mediators, and one of two functional outcome measures (academic functioning and social problems) as the dependent variable. The total problem score was not included in models with social problems as the outcome due to overlap with social problems. Secondary analyses were performed similarly, except they included the NIH Cognition Toolbox Composite (Luciana et al., 2018) raw score (an IQ measure) as a covariate, as previous research indicates grades (Roth et al., 2015) and social functioning (Almat, Aliya, Zhanna, & Gulmira, 2023) may be influenced by IQ. A third set of analyses examined weekday screen time instead of weekend screen time. Independent variables were taken from 1-year follow-up data, mediators from 2-year follow-up (except for diet, which was only available at 1-year follow-up), and outcome measures from 3-year follow-up questionnaires.

Significance of mediation models was determined by calculating the 95% confidence interval (CI) of the indirect effect based on 5,000 bias-corrected bootstrapped samples; an indirect effect in which the corresponding 95% CI was entirely positive or negative implies that *p* < .05. Percent mediation was also calculated using the indirect effect for each individual mediator as well as across all mediators combined by the equation *indirect effect* ÷ *total effect* × 100. Significant indirect effects were only considered to be of interest if the total effect (between the independent and dependent variable, including the mediator) was significant (*p* < .05).

### Moderated mediation effects

Effects of environmental moderators were examined using PROCESS v.5.0. A diagram of the analysis strategy for environmental moderation effects is shown in Supplementary Figure S1. These moderated mediation models examined the degree to which each moderator (e.g. family financial adversity) affected the mediation of associations between mental health and outcome by affecting the relationships between lifestyle factors and outcome (i.e. the mediation model ‘b’ path). We examined the moderation of the ‘b’ path (i.e. the path linking lifestyle factors to outcome), as the goal of this study was to examine everyday lifestyle mediators (e.g. screen time habits) that can be altered independent of a clinical provider. Consequently, we were particularly interested to examine how the beneficial effects of these mediators might be influenced by living conditions. Similar to the primary (nonmoderated) mediation analyses, significant moderated mediation was determined by calculating the 95% confidence interval (CI) of the index of moderated mediation based on 5,000 bias-corrected bootstrapped samples. Moderation effects (highest order unconditional interactions) were FDR-corrected for multiple comparisons (Benjamini & Hochberg, 1995) across all symptom domains for each moderator (e.g. for models predicting academic functioning, *p*-values were FDR-corrected for 16 comparisons [4 domains × 4 mediators]) for each moderator.

## Results

### Sample

Demographic, clinical, behavioral, environmental, and functional information for children included in the study are presented in Table 1. Briefly, the analyses with academic functioning as the outcome included 6,754 participants (after excluding siblings and children with missing data), and the analyses with social problems as the outcome included 7,550 participants. In all, 798 youth were missing academic functioning data, and 2 were missing social problems data. Comparing children with versus without academic functioning data suggested that those with missing data had higher symptoms and more screen time hours, although effect sizes for these effects were small (all Cohen’s *D* values < .20 except for weekday screen time, for which Cohen’s *D* = −.25) (Supplementary Table S1). Additionally, no difference in sex ratio was observed between youth with versus without missing data; for age, although statistically significant, the difference was qualitatively negligible (11.99 years vs. 12.09 years).

**Table 1.** Demographic, clinical, behavioral, environmental, and functional information for all participants included in analyses Table 1. long description.

Analysis-dependent variable	Grades in school, years 2–3	CBCL Social Problems score, year 3
*N*	6,754	7,550
*Demographic covariates*		
Age in years at Year 2	11.99 (.66)	12.00 (.66)
Sex *n* M/F/Intersex	3,578/3,174/2	3,988/3,560/2
NIH Cognition Toolbox Total Composite Raw Score, Baseline	87.11 (8.89)	86.79 (9.01)
*Clinical, independent (predictor) variables*		
CBCL Depression T-Score, Year 1	53.87 (5.97)	53.97 (6.08)
CBCL Anxiety T-Score, Year 1	53.55 (6.10)	53.63 (6.25)
PQ-BC Distress Score, Year 1	4.40 (9.05)	4.48 (9.09)
CBCL Total Problems T-Score, Year 1	45.51 (10.95)	45.65 (11.14)
*Healthy lifestyle mediators*		
Sleep Disturbances Score, Year 2	36.43 (8.01)	36.48 (8.06)
Mediterranean Diet Score, Year 1	8.08 (2.39)	8.07 (2.42)
Youth reported days physically active in the last week, Year 2 Questionnaire	3.80 (2.15)	3.78 (2.16)
Weekend screen time hours, Year 2	4.63 (3.07)	4.69 (3.13)
Weekday screen time hours, Year 2	3.30 (3.08)	3.38 (3.15)
*Environmental moderators*		
Neighborhood Crime Rating, Year 2	3.88 (.93)	3.87 (.94)
Family Conflict Score, Year 2	2.36 (1.94)	2.38 (1.96)
Youth Reported School Environment Score, Year 2	19.63 (2.76)	19.63 (2.78)
Family Financial Adversity Score, Baseline	.40 (1.01)	.42 (1.05)
*Functional, dependent (outcome) variables*		
Grades in school^a^, Years 2–3	3.73 (2.31)	–
CBCL Social Problems Score, Year 3	–	52.65 (4.90)

*Note:* Values are from parental report data unless otherwise specified (parental report data were used unless unavailable). Numbers in parentheses represent the standard deviation.

a A grade score of 3 corresponds to a GPA of 3.5–3.69 (A−) and a score of 4 to a GPA of 3.30–3.49 (B+). CBCL, Child Behavior Checklist; NIH, National Institutes of Health; PQ-BC, Prodromal Questionnaire-Brief Child Version.

### Mediation analyses: Mental health predicting academic functioning

**Figure 1.**
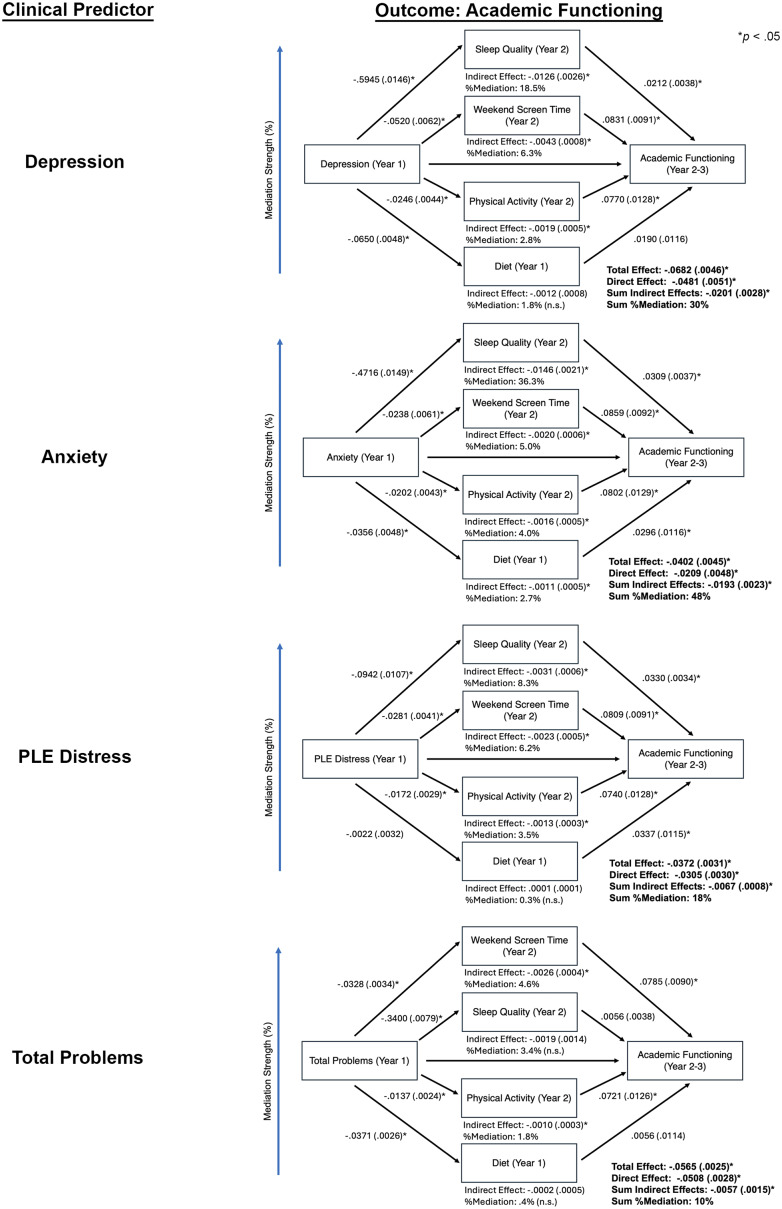
Results of mediation models predicting academic functioning (school grades between 2 and 3 year follow-up) while including age (2-year follow-up), sex, and site as covariates. More detailed results are provided in Supplementary Table S2. PLE, psychotic-like experiences. Figure 1. long description.

First, we ran a set of mediation analyses in which a mental health measure (depression, anxiety, distressing PLEs, and CBCL total problems) was the independent variable, four healthy lifestyle factors (sleep quality, weekend screen time, diet, and physical activity) were mediators, sex/age/site were covariates, and academic functioning (school grades) was the outcome. When entered simultaneously, these lifestyle factors combined significantly mediated associations between all four mental health variables with academic functioning (see Figure 1 for mediation models and Supplementary Table S2 for detailed statistics). The relationship between anxiety and academic functioning showed the highest mediation from these lifestyle mediators (48%), followed by depression (30%), distressing PLEs (18%), and finally total problems (10%). Furthermore, in three of the four models, sleep showed evidence of being the most important mediator, showing 36.3% (of 48% total) mediation for anxiety, 18.5% (of 30% total) mediation for depression, and 8.3% (of 18% total) mediation for distressing PLEs (Figure 1 and Supplementary Table S2). For total problems, screen time was the most important mediator (4.6%). Sleep was the second strongest mediator (3.4%) for total problems, but its mediation effects were nonsignificant. Screen time was usually the second-most important mediator, although its contributions were often noticeably less than sleep quality (e.g. 36.3% and 18.5% mediation by sleep quality vs. 5.0% and 6.3% mediation by screen time when anxiety and depression were the independent variables, respectively). Exercise also showed significant mediation, although its effects were even smaller than for screen time (e.g. 2.8% and 4.0% mediation for models predicting anxiety and depression, respectively). Diet did not show significant mediation for depression, distressing PLEs, or total problems.

Including cognition as a covariate did not appreciably alter the results of mediation models with academic functioning as the outcome (Supplementary Figure S2 and Supplementary Table S3). When weekday screen time was used instead of weekend screen time, the mediating effects of screen time were consistently smaller (e.g. 3.7% [weekday] vs.6.3% [weekend] for depression), although it remained statistically significant for all four psychopathological domains tested (Supplementary Figure S3 and Supplementary Table S4).

### Mediation analyses: Mental health predicting social functioning

**Figure 2.**
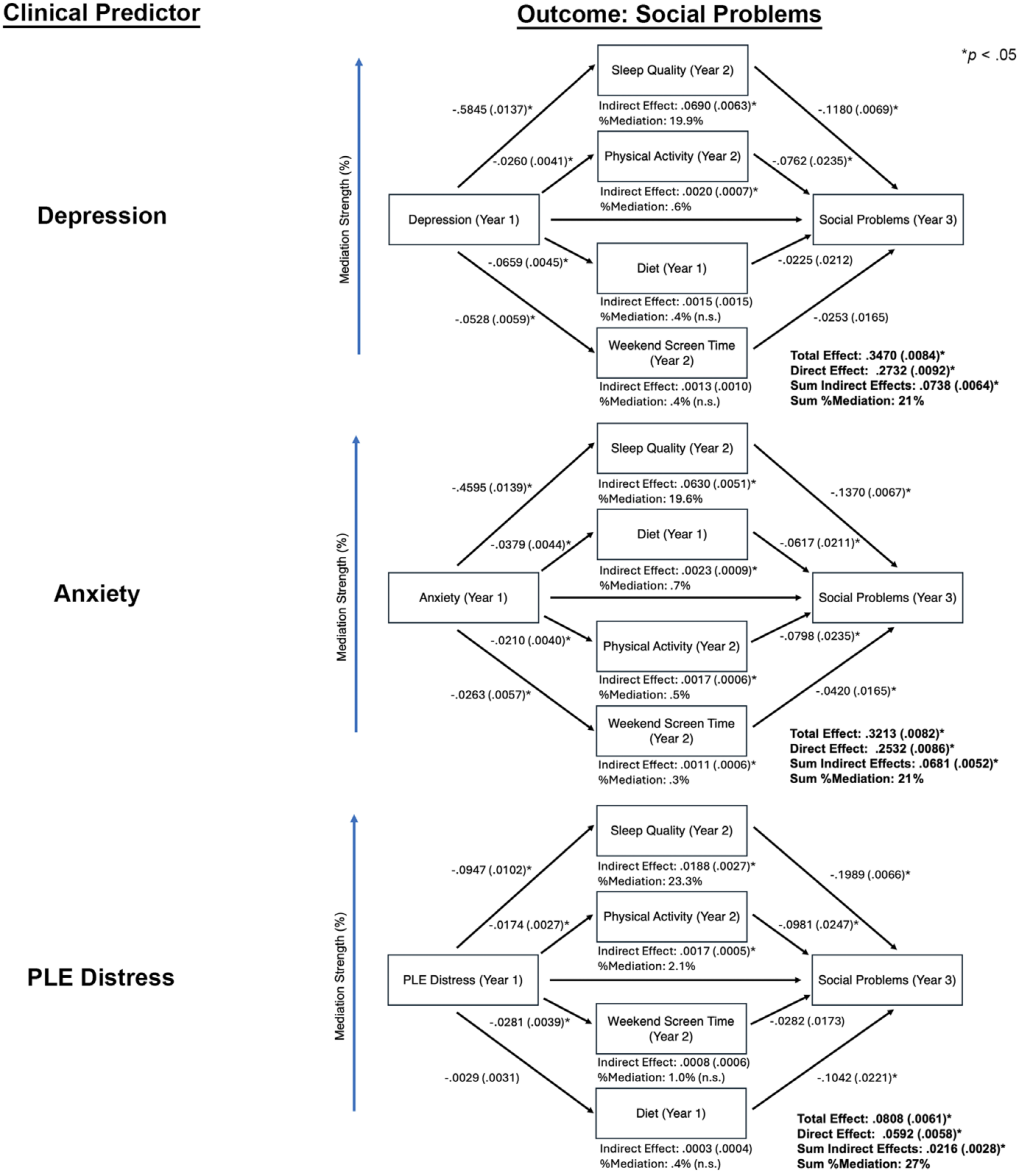
Results of mediation models predicting social problems (3 year CBCL Social Problems score) while including age (2-year follow-up), sex, and site as covariates. More detailed results are provided in Supplementary Table S5. PLE, psychotic-like experiences. Figure 2. long description.

Next, we ran a set of mediation analyses in which a mental health measure was the independent variable, the four healthy lifestyle factors were mediators, sex/age/site were covariates, and social problems (CBCL Social Problems score) were the outcome. As was the case for academic functioning, the four lifestyle mediators combined to show significant mediation in all psychopathological models (depression, anxiety, and distressing PLEs; Figure 2 and Supplementary Table S5). The relationship between distressing PLEs and social problems showed the highest mediation from these lifestyle mediators (27%), followed by depression (21%) and anxiety (21%). Strikingly, among the lifestyle mediators, only sleep quality was a robust, consistently significant mediator for all models examined. More specifically, for depression, sleep showed 19.9% (of 21% total) mediation; for anxiety, sleep showed 19.6% (of 21% total) mediation; and for PLE distress, sleep showed 23.3% (of 27% total) mediation. Thus, almost all of the healthy lifestyle mediation effects were driven by sleep quality for all three mental health domains examined.

Including cognition as a covariate did not appreciably alter the results of mediation models with social functioning as the outcome (Supplementary Figure S4 and Supplementary Table S6). When weekday screen time was used instead of weekend screen time, the mediating effects of screen time became consistently smaller and nonsignificant for all psychopathological domains tested (Supplementary Figure S5 and Supplementary Table S7).

### Environmental moderation effects

Supplementary Table S8 depicts how environmental factors moderate associations between lifestyle mediators with academic functioning, as well as moderator effects on percent mediation. The effects of significant moderators on mediation effects are further illustrated in Table 2. Specifically, as financial adversity increased, the mediating role of sleep quality and/or weekend screen time (reverse-scored) on associations between mental health and academic functioning decreased (Table 2).

**Table 2.** Changes in %mediation dependent on moderator level for significant moderators for models predicting academic functioning between years 2 and 3 Table 2. long description.

Predictor	Moderator	Moderator score	Indirect effect (95% CI)	%Mediation
Mediator				
*Depression*				
Sleep quality	Family financial adversity	Mean − 1 SD^a^	−.0138 (−.0193 to −.0084)	20.2%
		Mean	−.0120 (−.0172 to −.0070)	17.6%
		Mean + 1 SD	−.0076 (−.0139 to −.0015)	11.1%
Weekend screen time (reverse-scored**)**	Family financial adversity	Mean − 1 SD^a^	−.0045 (−.0063 to −.0030)	6.6%
		Mean	−.0040 (−.0056 to −.0027)	5.9%
		Mean + 1 SD	-.0027 (−.0043 to −.0015)	4.0%
*Anxiety*				
Sleep quality	Family financial adversity	Mean − 1 SD^a^	−.0153 (−.0199 to −.0112)	38.0%
		Mean	−.0138 (−.0181 to −.0101)	34.3%
		Mean + 1 SD	−.0102 (−.0153 to −.0055)	25.4%
Weekend screen time (reverse-scored)	Family financial adversity	Mean − 1 SD^a^	−.0021 (−.0035 to −.0010)	5.2%
		Mean	−.0019 (−.0031 to −.0009)	4.7%
		Mean + 1 SD	−.0013 (−.0025 to −.0006)	3.2%
*PLE distress*				
Sleep quality	Family financial adversity	Mean − 1 SD^a^	−.0032 (−.0047 to −.0022)	8.6%
		Mean	−.0029 (−.0043 to −.0020)	7.8%
		Mean + 1 SD	−.0022 (−.0035 to −.0013)	5.9%
Weekend screen time (reverse-scored)	Family financial adversity	Mean − 1 SD^a^	−.0023 (−.0035 to −.0015)	6.2%
		Mean	−.0021 (−.0032 to −.0013)	5.6%
		Mean + 1 SD	−.0015 (−.0025 to −.0008)	4.0%
*Total problems*				
Weekend screen time (reverse-scored)	Family financial adversity	Mean − 1 SD^a^	−.0027 (−.0038 to −.0019)	4.0%
		Mean	−.0024 (−.0034 to −.0017)	3.5%
		Mean + 1 SD	−.0016 (−.0026 to −.0009)	2.3%

*Note: See Table 1 for the mean (SD) of moderator variables.*

a Because mean − 1 SD < 0, mediation effects at this level were calculated when the moderator = 0.

Supplementary Table S9 depicts how environmental factors moderate associations among lifestyle mediators, with social problems as the outcome. The effects of significant moderators on mediation effects are further illustrated in Table 3. Specifically, as family conflict and financial adversity increased and the school environment worsened, the mediating effects of sleep quality on associations between mental health and social problems increased (Table 3).

**Table 3.** Changes in %mediation dependent on moderator level for significant moderators for models predicting social problems (CBCL Social Problems Score) at Year 3 Table 3. long description.

Predictor	Moderator	Moderator score	Indirect effect *B* (95% CI)	%Mediation
Mediator				
*Depression*				
Sleep quality	Family conflict	Mean − 1 SD	.0455 (.0318–.0603)	13.1%
		Mean	.0595 (.0484–.0716)	17.1%
		Mean + 1 SD	.0735 (.0593–.0890)	21.2%
Sleep quality	School environment	Mean − 1 SD	.0777 (.0632–.0933)	22.4%
		Mean	.0667 (.0549–.0795)	19.2%
		Mean + 1 SD	.0557 (.0408–.0719)	16.1%
Sleep quality	Family financial adversity	Mean − 1 SD^a^	.0590 (.0463–.0717)	17.0%
		Mean	.0639 (.0523–.0761)	18.4%
		Mean + 1 SD	.0762 (.0617–.0928)	22.0%
*Anxiety*				
Sleep quality	Family conflict	Mean − 1 SD	.0443 (.0332–.0554)	13.8%
		Mean	.0547 (.0457–.0648)	17.0%
		Mean + 1 SD	.0651 (.0539–.0777)	20.3%
Sleep quality	School environment	Mean − 1 SD	.0690 (.0576–.0816)	21.5%
		Mean	.0613 (.0519–.0719)	19.1%
		Mean + 1 SD	.0536 (.0419–.0673)	16.7%
Sleep quality	Family financial adversity	Mean − 1 SD^a^	.0543 (.0451–.0650)	16.9%
		Mean	.0584 (.0494–.0685)	18.2%
		Mean + 1 SD	.0683 (.0564–.0821)	21.3%
*PLE distress*				
Sleep quality	Family conflict	Mean − 1 SD^a^	.0139 (.0010–.0188)	17.2%
		Mean	.0165 (.0121–.0215)	20.4%
		Mean + 1 SD	.0191 (.0140–.0251)	23.6%
Sleep quality	School environment	Mean − 1 SD^a^	.0202 (.0151–.0265)	25.0%
		Mean	.0184 (.0137–.0240)	22.8%
		Mean + 1 SD	.0167 (.0120–.0223)	20.7%
Sleep quality	Family financial adversity	Mean − 1 SD^a^	.0172 (.0126–.0224)	21.3%
		Mean	.0179 (.0133–.0233)	22.2%
		Mean + 1 SD	.0196 (.0146–.0257)	24.3%

*Note: See Table 1 for the mean (SD) of moderator variables.*

a Because mean − 1 SD < 0, mediation effects at this level were calculated when the moderator = 0.

## Discussion

The present study examined the degree to which potentially modifiable lifestyle behaviors, including sleep quality, physical activity, diet quality, and screen time, serve as mediators linking adolescent mental health symptoms to academic functioning and social problems, as well as whether these associations were moderated in the context of environmental adversity. Using data from the ABCD Study, we found evidence that healthy lifestyle factors, especially sleep, mediated associations between depression, anxiety, and PLEs with both academic and social functioning. We also found evidence that financial adversity modified associations between lifestyle factors with both academic and social problems, and that school environment and family conflict additionally modified associations with social problems. These findings clarify modifiable pathways that may contribute to functional decline in adolescence and identify contextual conditions under which lifestyle-focused interventions may be less effective.

### Lifestyle mediators linking symptoms to academic functioning

Consistent with predictions, healthy lifestyle factors jointly mediated the associations between symptoms and academic functioning across all mental health domains examined. For academic functioning, mediation was strongest for models using anxiety as the predictor (48% of the total effect), followed by depression, PLEs, and total mental health concerns. This pattern may suggest that academic functioning is particularly sensitive to internalizing processes (e.g. cognitive rumination and worrying), domains that may be especially intertwined with lifestyle behaviors (Dalforno, Wengert, Kim, & Jacobsen, 2022; Fergusson & Woodward, 2002; Finning et al., 2019a, 2019b). Sleep quality emerged as the dominant mediator across all analyses (i.e. explaining the majority of the total mediated effect), with screen time providing additional contributions. These findings point to sleep quality as an important factor through which early symptoms translate into reduced academic performance, consistent with evidence linking sleep irregularities to daytime fatigue, attention problems, and poorer executive functioning (Dalforno, Wengert, Kim, & Jacobsen, 2022; Fergusson & Woodward, 2002; Finning et al., 2019a, 2019b; Kearney, Dupont, Fensken, & Gonzálvez, 2023). Additionally, screen time showed a moderate influence, while exercise and diet showed less robust mediation effects, with their small but significant contributions in some models. This highlights the value of a multi-behavioral approach to supporting academic outcomes (Camprodon-Boadas et al., 2025; Firth et al., 2020; Green et al., 2013; O’Neil et al., 2014; Ruiz-Ranz & Asin-Izquierdo, 2025). As effect sizes for screen time, diet, and exercise were small (<7%), making changes to these three lifestyle factors is unlikely to have a dramatic effect on outcomes. The observational nature of our study also cannot rule out the possibility of bidirectional effects (e.g. mental health influencing sleep quality instead of vice versa). Overall, however, these results suggest that interventions that improve sleep quality and/or (to a lesser extent) restrict screen time may reduce the deleterious effects of mental health problems on academic functioning.

As an aside, it is notable that weekend screen time showed a qualitatively stronger mediating effect than weekday screen time. One possible reason for the discrepancy is that the dynamic range for weekday screen time is lower (as children are in school, and as evidenced by the lower mean weekday screen time; Table 1), reducing its ability to mediate functional outcomes.

### Lifestyle mediators linking symptoms to social problems

When social problems were the outcome, a similar overall mediation pattern emerged as compared to academic functioning, although the strength of the mediation differed. Mediation was strongest for PLE distress (27%), consistent with some psychosis spectrum research (El Bouhaddani et al., 2018; Huckle, Lemmel, & Johnson, 2021; Steenkamp et al., 2021), followed by depression and anxiety (both ~21%) (Achterbergh et al., 2020; Kupferberg & Hasler, 2023). Unlike the academic functioning models, where multiple healthy lifestyle factors contributed, sleep quality was the only consistently significant mediator of social functioning across all domains. This may indicate that sleep plays a uniquely central role in how symptoms translate into impairments in social functioning. This is potentially consistent with previous research linking mental health concerns, including social anhedonia and irritability, with social problems (Qiu & Morales-Munoz, 2022; Woodfield, Butler, & Tsappis, 2024). In contrast, screen time, diet, and physical activity contributed minimally to social problems pathways, suggesting these behaviors exert a smaller influence on associations between mental health and social problems. The contrast between academic functioning and social problems mediation patterns underscores that healthy lifestyle factors may operate differently across functional domains, with sleep exerting a more domain-general influence, and other lifestyle factors (e.g. screentime) playing more domain-specific roles. As with academic functioning, these results also suggest that interventions that improve sleep quality may help mitigate the effects of poor mental health on social outcomes.

### Moderated mediation by environmental adversity

Environmental moderation analyses revealed that the pathways described above were not uniform across youth. For example, for academic functioning models, financial adversity was a significant moderator in that higher financial adversity weakened associations between sleep quality and the academic outcome in these models. This may suggest that, in the context of financial strain, lifestyle improvements may confer smaller academic functioning benefits. This may be because economic stressors more directly affect academic functioning (e.g. through resource limitations and unstable housing), overshadowing lifestyle-related influences (Gautam, Dessie, Rahman, & Khanam, 2023; Gautam, Rahman, & Khanam, 2025).

Interestingly, a model related to this hypothesis was tested in a 2007 study by Buckhalt, El-Sheikh, and Keller (2007). This study analyzed socioeconomic moderators of the relationship between sleep quality and cognitive performance in children aged 7–11. Buckhalt, El-Sheikh, and Keller (2007) found that socioeconomic status moderated relationships between sleep quality and intellectual ability. The authors reported that longer sleep duration was related to greater processing speed and better working memory in children of higher, but not lower, socioeconomic status. Interestingly, Buckhalt, El-Sheikh, and Keller (2007) also found that bedroom space may have been a significant contributor to this effect, as children of lower socioeconomic status were more likely to share a bedroom. Mirroring our hypothesis above, Buckhalt, El-Sheikh, and Keller (2007) also speculated that stressors associated with low socioeconomic status (independent of those associated with sleep) could be driving the observed moderation effects. As this hypothesis is speculative, however, it requires further investigation.

For social problems, the pattern of moderated mediation differed from that observed for models examining academic functioning. Whereas financial adversity weakened lifestyle-related mediation for academic functioning, the opposite was true for social problems: as family conflict and financial adversity increased and the school environment worsened, the mediating effects of sleep quality on associations between mental health and social problems strengthened. One possibility is that, under high-conflict or high-adversity conditions, sleep disruptions may exacerbate emotional reactivity, irritability, or social withdrawal, thereby magnifying their downstream impact on social functioning (Achterbergh et al., 2020; Cummings, Koss, & Davies, 2015). Taken together, these findings indicate that healthy lifestyle factors operate differently across functional domains, with sleep quality becoming a stronger mediator of social functioning under higher adversity, in contrast to the diminished mediation observed for academic functioning.

### Implications for prevention and intervention

This study identifies sleep quality and, to a lesser extent, screen time as potentially tractable intervention targets for improving outcomes among adolescents experiencing mental health concerns. Sleep interventions, such as school-based sleep hygiene programs to improve sleep quality, may meaningfully improve academic and social functioning (Fenwick-Smith, Dahlberg, & Thompson, 2018; Green et al., 2013). Yet, the moderating effects of financial adversity and family conflict suggest that lifestyle-focused interventions should be tailored to the youth’s environmental context. For youth experiencing economic hardship or high family conflict, sleep-based supports may need to be paired with additional structural supports (e.g. resource assistance and family-based therapies) to maximize impact.

### Limitations

Despite the strengths of the present study, several limitations warrant consideration. First, although the present work focused on modifiable lifestyle variables, other lifestyle factors may likely influence both mental health and social and academic functioning. Second, even though mediation effects were statistically robust, several of the effect sizes were modest and may not generalize uniformly across developmental periods. Third, while sleep problems are a diagnostic feature of several mental health conditions (e.g. mood disorders), it is notable that sleep disturbances partially mediated associations with a range of mental health symptoms. This suggests sleep may function as a separable and potentially modifiable factor, rather than intrinsic to experiencing specific symptoms such as depression. Fourth, sleep, screen time, and academic functioning information were taken from parent reports, which may have inflated associations due to shared method variance. Fifth, it is possible that differences in the effect size of the mediators were affected by measurement precision, as the number of questions that were combined to result in a score for each mediator differed (e.g. one question for physical activity vs. 26 questions for sleep quality). Finally, although mediation models incorporated independent variables, mediators, and outcomes in chronological order (except for diet, as this information was only available for a 1-year follow-up, and would be unlikely to change by 2-year follow-up; da Costa, Severo, Araujo, & Vilela, 2024), this does not necessarily imply that these relationships were causal. This is because mediator and outcome data from earlier time points were not considered in mediation models, making it possible that these findings reflect stable between-person differences rather than longitudinal within-person processes. Related to this point and as stated previously, because our study is observational (i.e. does not test the effect of an intervention), it does not rule out the possibility that mediation effects are bi-directional (e.g. symptoms causing sleep problems instead of vice versa). Future studies using more sophisticated structural equation models incorporating data from multiple time points are necessary to examine the longitudinal relationships between these factors more comprehensively, and intervention studies are necessary to firmly establish cause-and-effect relationships. This may necessitate using data from future ABCD studies, as later releases will include more time points (including data during middle and late adolescence, when mental issues become increasingly prevalent (Substance Abuse and Mental Health Services Administration, 2013. Behavioral Health, United States, 2012. HHS Publication No. (SMA) 13-4797. Rockville, MD: Substance Abuse and Mental Health Services Administration).

## Conclusions

Findings from this large, multisite study provide novel evidence that lifestyle factors, especially sleep quality, are key factors linking adolescent mental health symptom domains to academic and social functioning. These pathways are sensitive to contextual conditions, with financial adversity and family conflict moderating the impact of lifestyle-related mediation. Together, these results underscore the importance of integrating lifestyle-based and family-informed approaches to support adolescents experiencing early mental health concerns.

## Supporting information

10.1017/S0033291726105121.sm001 Smucny et al. supplementary material Smucny et al. supplementary material

## Supplementary material

The supplementary material for this article can be found at http://doi.org/10.1017/S0033291726105121.

## Long descriptions

### Table 1. Long description

The table is organized into five thematic sections.

1. Sample Size: N equals 6,754 for the Grades group and 7,550 for the CBCL group.

2. Demographic Covariates:

- Age in years at Year 2: 11.99 for Grades, 12.00 for CBCL.

- Sex (Male/Female/Intersex): 3,578/3,174/2 for Grades, 3,988/3,560/2 for CBCL.

- NIH Cognition Toolbox Total Composite Raw Score: 87.11 for Grades, 86.79 for CBCL.

3. Clinical Independent Variables (Year 1):

- CBCL Depression T-Score: 53.87 for Grades, 53.97 for CBCL.

- CBCL Anxiety T-Score: 53.55 for Grades, 53.63 for CBCL.

- PQ-BC Distress Score: 4.40 for Grades, 4.48 for CBCL.

- CBCL Total Problems T-Score: 45.51 for Grades, 45.65 for CBCL.

4. Healthy Lifestyle Mediators:

- Sleep Disturbances Score (Year 2): 36.43 for Grades, 36.48 for CBCL.

- Mediterranean Diet Score (Year 1): 8.08 for Grades, 8.07 for CBCL.

- Days physically active (Year 2): 3.80 for Grades, 3.78 for CBCL.

- Weekend screen time hours (Year 2): 4.63 for Grades, 4.69 for CBCL.

- Weekday screen time hours (Year 2): 3.30 for Grades, 3.38 for CBCL.

5. Environmental Moderators:

- Neighborhood Crime Rating (Year 2): 3.88 for Grades, 3.87 for CBCL.

- Family Conflict Score (Year 2): 2.36 for Grades, 2.38 for CBCL.

- Youth Reported School Environment Score (Year 2): 19.63 for both groups.

- Family Financial Adversity Score (Baseline): 0.40 for Grades, 0.42 for CBCL.

6. Functional Dependent Outcome Variables:

- Grades in school (Years 2-3): 3.73 for the Grades group.

- CBCL Social Problems Score (Year 3): 52.65 for the CBCL group.

Navigate back to Table 1..

### Figure 1. Long description

The diagram contains four panels, each representing a mediation model with a Clinical Predictor on the left, four Mediators in the center, and Academic Functioning (Year 2-3) on the right.

1. Depression Model: Depression (Year 1) predicts Academic Functioning. Mediators include Sleep Quality (Year 2), Weekend Screen Time (Year 2), Physical Activity (Year 2), and Diet (Year 1). Sleep Quality shows the highest mediation strength at 18.5 percent. The total effect is minus .0682 and the sum of indirect effects is minus .0201.

2. Anxiety Model: Anxiety (Year 1) is the predictor. Sleep Quality is the strongest mediator at 36.3 percent. The total effect is minus .0402 and the sum of indirect effects is minus .0193.

3. P L E Distress Model: P L E Distress (Year 1) is the predictor. Sleep Quality mediates 8.3 percent and Weekend Screen Time mediates 6.2 percent. The total effect is minus .0372 and the sum of indirect effects is minus .0067.

4. Total Problems Model: Total Problems (Year 1) is the predictor. Weekend Screen Time is the primary mediator at 4.6 percent, followed by Sleep Quality at 3.4 percent. The total effect is minus .0565 and the sum of indirect effects is minus .0057.

In all models, arrows indicate the direction of influence with associated beta coefficients and standard errors. Asterisks indicate significance at p less than .05. A vertical blue arrow to the left of each model indicates Mediation Strength percentage.

Navigate back to Figure 1..

### Figure 2. Long description

The diagram consists of three stacked panels representing mediation models for Depression, Anxiety, and P L E Distress. Each panel follows a structure where a Year 1 predictor on the left connects via arrows to four mediators in the center, which then connect to Social Problems Year 3 on the right.

Top Panel: Depression Year 1.

* Mediators from top to bottom: Sleep Quality Year 2, Physical Activity Year 2, Diet Year 1, and Weekend Screen Time Year 2.

* Sleep Quality shows the highest mediation strength at 19.9 percent.

* Total Effect: .3470. Direct Effect: .2732. Sum of Indirect Effects: .0738.

Middle Panel: Anxiety Year 1.

* Mediators from top to bottom: Sleep Quality Year 2, Diet Year 1, Physical Activity Year 2, and Weekend Screen Time Year 2.

* Sleep Quality shows the highest mediation strength at 19.6 percent.

* Total Effect: .3213. Direct Effect: .2532. Sum of Indirect Effects: .0681.

Bottom Panel: P L E Distress Year 1.

* Mediators from top to bottom: Sleep Quality Year 2, Physical Activity Year 2, Weekend Screen Time Year 2, and Diet Year 1.

* Sleep Quality shows the highest mediation strength at 23.3 percent.

* Total Effect: .0808. Direct Effect: .0592. Sum of Indirect Effects: .0216.

Statistical notes: Asterisks indicate p < .05. Values on arrows represent path coefficients and standard errors. Vertical blue arrows on the left of each panel indicate Mediation Strength percentage increasing from bottom to top.

Navigate back to Figure 2..

### Table 2. Long description

The table is organized into four main sections based on the predictor: Depression, Anxiety, P L E distress, and Total problems. For all models, the moderator is Family financial adversity, evaluated at three levels: Mean minus 1 S D, Mean, and Mean plus 1 S D.

1. Depression section:

- Sleep quality mediator: Indirect effect decreases from minus .0138 to minus .0076 as adversity increases; percentage mediation drops from 20.2 percent to 11.1 percent.

- Weekend screen time mediator: Indirect effect decreases from minus .0045 to minus .0027; percentage mediation drops from 6.6 percent to 4.0 percent.

2. Anxiety section:

- Sleep quality mediator: Indirect effect decreases from minus .0153 to minus .0102; percentage mediation drops from 38.0 percent to 25.4 percent.

- Weekend screen time mediator: Indirect effect decreases from minus .0021 to minus .0013; percentage mediation drops from 5.2 percent to 3.2 percent.

3. P L E distress section:

- Sleep quality mediator: Indirect effect decreases from minus .0032 to minus .0022; percentage mediation drops from 8.6 percent to 5.9 percent.

- Weekend screen time mediator: Indirect effect decreases from minus .0023 to minus .0015; percentage mediation drops from 6.2 percent to 4.0 percent.

4. Total problems section:

- Weekend screen time mediator: Indirect effect decreases from minus .0027 to minus .0016; percentage mediation drops from 4.0 percent to 2.3 percent.

Note: All indirect effects include 95 percent confidence intervals in parentheses. Mediation effects for Mean minus 1 S D were calculated at zero when the value was less than zero.

Navigate back to Table 2..

### Table 3. Long description

The table is divided into three main sections based on the predictor: Depression, Anxiety, and PLE distress. All models use Sleep quality as the mediator.

1. Depression Section:

- Family conflict moderator: Percent mediation increases from 13.1 percent at Mean minus 1 S D to 21.2 percent at Mean plus 1 S D.

- School environment moderator: Percent mediation decreases from 22.4 percent at Mean minus 1 S D to 16.1 percent at Mean plus 1 S D.

- Family financial adversity moderator: Percent mediation increases from 17.0 percent at Mean minus 1 S D to 22.0 percent at Mean plus 1 S D.

2. Anxiety Section:

- Family conflict moderator: Percent mediation increases from 13.8 percent at Mean minus 1 S D to 20.3 percent at Mean plus 1 S D.

- School environment moderator: Percent mediation decreases from 21.5 percent at Mean minus 1 S D to 16.7 percent at Mean plus 1 S D.

- Family financial adversity moderator: Percent mediation increases from 16.9 percent at Mean minus 1 S D to 21.3 percent at Mean plus 1 S D.

3. P L E distress Section:

- Family conflict moderator: Percent mediation increases from 17.2 percent at Mean minus 1 S D to 23.6 percent at Mean plus 1 S D.

- School environment moderator: Percent mediation decreases from 25.0 percent at Mean minus 1 S D to 20.7 percent at Mean plus 1 S D.

- Family financial adversity moderator: Percent mediation increases from 21.3 percent at Mean minus 1 S D to 24.3 percent at Mean plus 1 S D.

Indirect effect B values with 95 percent confidence intervals are provided for every row, showing positive correlations across all moderated mediation paths.

Navigate back to Table 3..
